# β-Funaltrexamine Displayed Anti-Inflammatory and Neuroprotective Effects in Cells and Rat Model of Stroke

**DOI:** 10.3390/ijms21113866

**Published:** 2020-05-29

**Authors:** Chih-Cheng Wu, Cheng-Yi Chang, Kuei-Chung Shih, Chih-Jen Hung, Ya-Yu Wang, Shih-Yi Lin, Wen-Ying Chen, Yu-Hsiang Kuan, Su-Lan Liao, Wen-Yi Wang, Chun-Jung Chen

**Affiliations:** 1 Department of Anesthesiology, Taichung Veterans General Hospital, Taichung City 407, Taiwan; chihcheng.wu@gmail.com (C.-C.W.); hung@vghtc.gov.tw (C.-J.H.); 2 Department of Financial Engineering, Providence University, Taichung City 433, Taiwan; 3 Department of Data Science and Big Data Analytics, Providence University, Taichung City 433, Taiwan; 4 Department of Surgery, Feng Yuan Hospital, Taichung City 420, Taiwan; c.y.chang.ns@gmail.com; 5 Department of Computer Science and Information Management, Providence University, Taichung City 433, Taiwan; kcshih0307@gmail.com; 6 Department of Family Medicine, Taichung Veterans General Hospital, Taichung City 407, Taiwan; yywang@vghtc.gov.tw; 7 Institute of Clinical Medicine, National Yang Ming University, Taipei City 112, Taiwan; sylin@vghtc.gov.tw; 8 Center for Geriatrics and Gerontology, Taichung Veterans General Hospital, Taichung City 407, Taiwan; 9 Department of Veterinary Medicine, National Chung-Hsing University, Taichung City 402, Taiwan; wychen@dragon.nchu.edu.tw; 10 Department of Pharmacology, Chung Shan Medical University, Taichung City 402, Taiwan; kuanyh001@gmail.com; 11 Department of Medical Research, Taichung Veterans General Hospital, Taichung City 407, Taiwan; slliao@vghtc.gov.tw; 12 Department of Nursing, Hung Kuang University, Taichung City 433, Taiwan; walice@sunrise.hk.edu.tw; 13 Department of Medical Laboratory Science and Biotechnology, China Medical University, Taichung City 404, Taiwan

**Keywords:** microglia polarization, neuroinflammation, opioid, stroke

## Abstract

Chronic treatment involving opioids exacerbates both the risk and severity of ischemic stroke. We have provided experimental evidence showing the anti-inflammatory and neuroprotective effects of the μ opioid receptor antagonist β-funaltrexamine for neurodegenerative diseases in rat neuron/glia cultures and a rat model of cerebral Ischemia/Reperfusion (I/R) injury. Independent of in vitro Lipopolysaccharide (LPS)/interferon (IFN-γ)-stimulated neuron/glia cultures and in vivo cerebral I/R injury in Sprague–Dawley rats, β-funaltrexamine downregulated neuroinflammation and ameliorated neuronal degeneration. Alterations in microglia polarization favoring the classical activation state occurred in LPS/IFN-γ-stimulated neuron/glia cultures and cerebral I/R-injured cortical brains. β-funaltrexamine shifted the polarization of microglia towards the anti-inflammatory phenotype, as evidenced by decreased nitric oxide, tumor necrosis factor-α, interleukin-1β, and prostaglandin E2, along with increased CD163 and arginase 1. Mechanistic studies showed that the suppression of microglia pro-inflammatory polarization by β-funaltrexamine was accompanied by the reduction of NF-κB, AP-1, cyclic AMP response element-binding protein, along with signal transducers and activators of transcription transcriptional activities and associated upstream activators. The effects of β-funaltrexamine are closely linked with its action on neuroinflammation by switching microglia polarization from pro-inflammatory towards anti-inflammatory phenotypes. These findings provide new insights into the anti-inflammatory and neuroprotective mechanisms of β-funaltrexamine in combating neurodegenerative diseases, such as stroke.

## 1. Introduction

Opioids are potent analgesics and commonly prescribed for the treatment of severe pain and for use in anesthesia. Dynorphins, enkephalins, and β-endorphins are members of the endogenous opioid peptide family which mediate their effects through three subtypes of G-protein coupled membrane receptors, named, μ, δ, and κ. The endogenous opioidergic system is widely distributed throughout the Central Nervous System (CNS) as well as the peripheral tissues. Beyond its effect on the nociceptive/analgesic systems, the opioidergic system also displays effects on physiological and behavioral functions, including respiration, ion channel activity, and immune responses [1,2]. Additionally, alteration in the opioidergic system has been reported in the disease initiation and progression of several acute and chronic diseases, including neurodegenerative diseases [3,4,5,6,7,8,9]. These phenomena highlight the potential pathogenic roles that the opioidergic system may have and candidate targets for intervention with an aim to prevent and/or treat diseases.

As with naturally occurring opioid peptides, the exogenous opioid peptide mimetic exerts the pharmacological effects through binding to specific opioid receptors. Upon binding with either the μ, δ, or κ opioid receptor, they display shared or distinct biological consequences. For example, the neuroprotective effects of the δ and κ opioid receptor agonists and μ opioid receptor antagonist have been described [7,9,10,11]. Despite morphine long being known to have immunosuppressive properties in vivo, a chronic morphine infusion causes neuroinflammation [12,13]. In vitro, both microglia and macrophages respond to morphine by releasing pro-inflammatory cytokines [14,15,16,17,18]. Because the well-known effects of morphine are mediated specifically by the μ opioid receptor subtype, the activation of the μ opioid receptor is closely linked with neuroinflammation. Accordingly, the μ opioid receptor agonists such as d- Ala^2^-MePhe^4^-Glyol^5^-Enkephalin (DAMGO) and fentanyl display pro-inflammatory potential in microglia and astrocytes, while the μ opioid receptor antagonists suppress immune cell activation and pro-inflammatory cytokine expression challenged with morphine, Lipopolysaccharide (LPS), ethanol, or fentanyl [15,17,19,20]. Parallel suppression in inflammatory responses is observed in the presence of δ and κ opioid receptor agonists against LPS or ethanol treatments [20,21,22,23]. Since neuroinflammation plays a substantial role in the pathogenesis of neurodegenerative diseases, the distinct biological functions towards neuroprotection amongst the three subtypes of opioid receptors seem to be closely linked with their effects on neuroinflammation.

The anti-inflammatory and neuroprotective effects of (−)-naloxone, a nonselective opioid receptor antagonist, have been described [11,24,25,26]. However, the anti-inflammatory effects of its inactive stereoisomer (+)-naloxone occur through an opioid receptor in an independent manner [3,18]. β-funaltrexamine is a selective μ opioid receptor antagonist. Increasing evidence suggests it has anti-inflammatory effects in LPS-stimulated human astrocytes and mice. However, it is ineffective in reversing μ opioid receptor agonist-induced pro-inflammatory responses [19,27,28,29,30]. These findings indicate that β-funaltrexamine and naloxone may have the potential to be developed as alternative anti-inflammatory agents.

Previously, we had reported on the neuroprotective effects of opioidergic agents, BW373U86 (δ agonist), dynorphin A 1-13 (κ agonist), and naloxone (nonselective antagonist) but not H-Tyr-d-Ala-Phe-Phe-NH_2_ (TAPP) (μ agonist) in rat model of cerebral Ischemia/Reperfusion (I/R) injury [11,24]. To extend the scope of β-funaltrexamine’s biological implications, its anti-inflammatory and neuroprotective identities were further explored in rat primary neuron/glia cultures, with the effects then extrapolated to in vivo evaluation in a rat model of cerebral I/R injury.

## 2. Results

### 2.1. β-Funaltrexamine Alleviated Neurotoxic Cytokine Production

**Figure 1:**
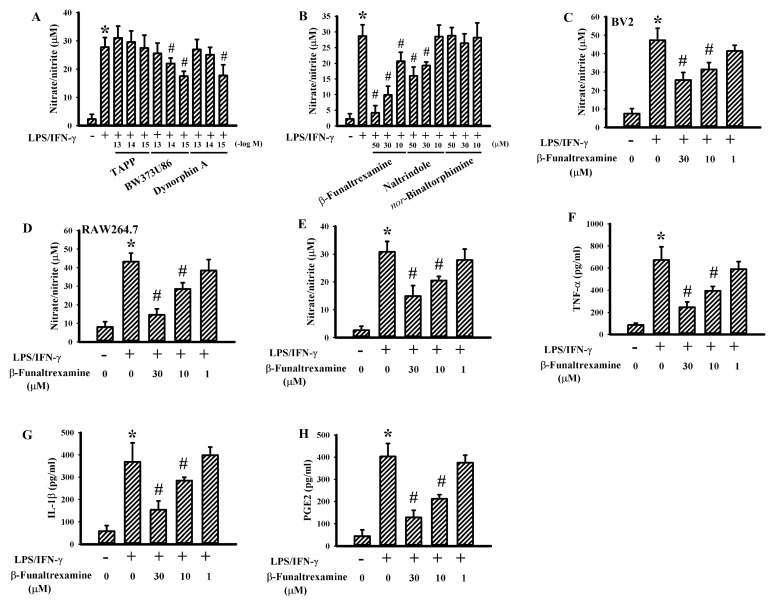
β-funaltrexamine alleviated neurotoxic cytokine production in neuron/glia cultures. Neuron/glia cultures were pretreated with vehicle, various concentrations of H-Tyr-d-Ala-Phe-Phe-NH_2_ (TAPP), BW373U86, dynorphin A (**A**), β-funaltrexamine, naltrindole, or *nor*-binaltorphimine (**B**) for 30 min before being incubated with Lipopolysaccharide (LPS) (100 ng/mL)/Interferon-gamma (IFN-γ) (10 U/mL) for an additional 24 h. BV2 cells (**C**) and RAW264.7 cells (**D**) were pretreated with vehicle or various concentrations of β-funaltrexamine for 30 min before being incubated with LPS (100 ng/mL)/IFN-γ (10 U/mL) for an additional 24 h. Supernatants were collected and subjected to Griess reagent for the measurement of NO. Neuron/glia cultures were pretreated with vehicle or various concentrations of β-funaltrexamine for 30 min before being incubated with LPS (100 ng/mL)/IFN-γ (10 U/mL) for an additional 24 h. Supernatants were collected and subjected to Griess reagent or ELISA for the measurement of NO (**E**), Tumor Necrosis Factor-α (TNF-α) (**F**), Interleukin-1β (IL-1β) (**G**), and Prostaglandin E2 (PGE2) (**H**). * *p* < 0.05 vs. untreated control and # *p* < 0.05 vs. LPS/IFN-γ control, *n* = 4.

To explore the anti-inflammatory potential of opioidergic compounds, neonatal rat neuron/glia cultures consisting of neurons, astrocytes, and microglia were prepared. LPS/interferon-gamma (IFN-γ) stimulation caused the release of elevated levels of Nitric Oxide (NO). Ultralow concentrations of the δ opioid receptor agonist BW373U86 and κ opioid receptor agonist Dynorphin A but not the μ opioid receptor agonist TAPP decreased LPS/IFN-γ-induced NO production (Figure 1A). A parallel reduction in NO release was observed in the presence of the μ opioid receptor antagonist β-funaltrexamine and δ opioid receptor antagonist naltrindole, while the κ opioid receptor antagonist *nor*-binaltorphimine experienced the least effects (Figure 1B). As with the neuron/glia cultures, the effects of β-funaltrexamine were duplicated in the murine BV2 microglial cell line (Figure 1C) and the RAW264.7 macrophage cell line (Figure 1D). Concurrently, β-funaltrexamine alleviated the production of NO (Figure 1E), Tumor Necrosis Factor-α (TNF-α) (Figure 1F), Interleukin-1β (IL-1β) (Figure 1G), and Prostaglandin E2 (PGE2) (Figure 1H) in LPS/IFN-γ-stimulated neuron/glia cultures as well. These findings indicate that there is an inhibitory effect of β-funaltrexamine on neurotoxic cytokine production.

### 2.2. β-Funaltrexamine Alleviated Neuronal Cell Death

**Figure 2:**
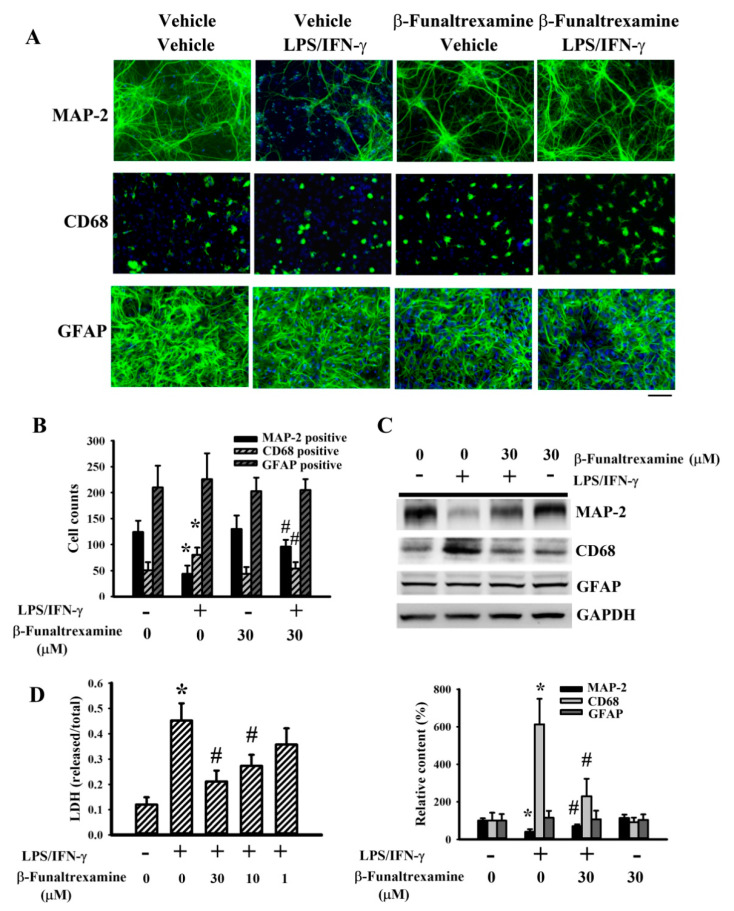
β-funaltrexamine alleviated neuronal cell death. Neuron/glia cultures were pretreated with vehicle or β-funaltrexamine (30 μM) for 30 min before being incubated with LPS (100 ng/mL)/IFN-γ (10 U/mL) for an additional 48 h. Cells were subjected to immunofluorescence staining with antibodies recognizing Microtubule-Associated Protein 2 (MAP-2), CD68, and Glial Fibrillary Acidic Protein (GFAP). The cell nuclei were counterstained with Hoechst 33342. Representative micrographs are shown. Bar = 60 μm (**A**). Quantitative numbers of immunopositivity are shown (**B**). Total cellular proteins were extracted and subjected to Western blot analysis with indicated antibodies. One representative blot of four independent culture batches is shown (**C**). Cell damage was measured by LDH efflux assay (**D**). * *p* < 0.05 vs. untreated control and # *p* < 0.05 vs. LPS/IFN-γ control, *n* = 4.

The production of neurotoxic cytokines simultaneously causes neuronal cell death in LPS/IFN-γ-treated neuron/glia cultures [31]. The morphological integrities of visible neurons, microglia, and astrocytes in neuron/glia cultures were examined by the immunoreactivity of Microtubule-Associated Protein 2 (MAP-2), CD68, and Glial Fibrillary Acidic Protein (GFAP) (Figure 2A), respectively. LPS/IFN-γ treatment caused neuronal cell degeneration and the disruption of neuronal cell integrity, while a reduction of visible neuronal cell numbers was alleviated by β-funaltrexamine (Figure 2A). Upon LPS/IFN-γ exposure, ramified microglia changed into reactive microglia of phagocytic morphology. The alternations in microglia morphology were alleviated by β-funaltrexamine (Figure 2A). However, the morphological integrity of astrocytes was not remarkably altered by LPS/IFN-γ or β-funaltrexamine (Figure 2A). Quantitative measurement of cell numbers in MAP-2-, CD68-, and GFAP-immunopositivity (Figure 2B) as well as protein contents in MAP-2, GFAP, and CD68 displayed the same results (Figure 2C) as those in immunofluorescence detection (Figure 2A). Cell damage was further examined by measuring Lactate Dehydrogenase (LDH) efflux. LPS/IFN-γ treatment caused an increase of LDH efflux, while β-funaltrexamine decreased the LDH efflux (Figure 2D). These findings indicate that LPS/IFN-γ induced selective neuronal cell death within neuron/glia cultures and that β-funaltrexamine showed neuroprotective effects.

### 2.3. β-Funaltrexamine Alleviated Microglia Activation

**Figure 3:**
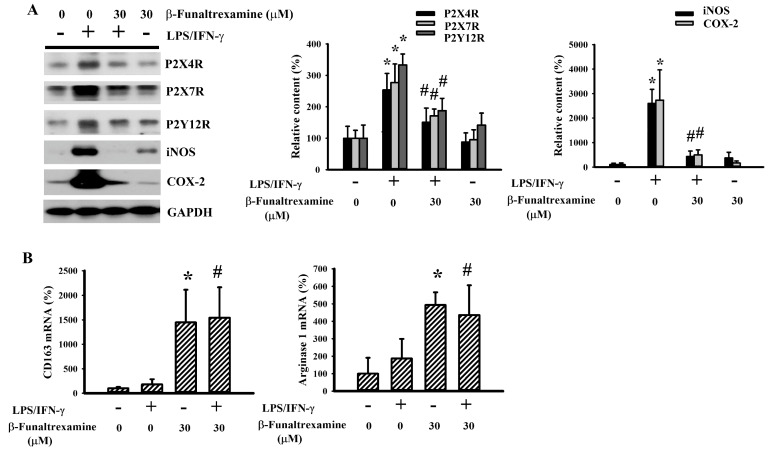
β-funaltrexamine alleviated microglial activation. Neuron/glia cultures were pretreated with vehicle or β-funaltrexamine (30 μM) for 30 min before being incubated with LPS (100 ng/mL)/IFN-γ (10 U/mL) for an additional 24 h. (**A**) Total cellular proteins were extracted and subjected to Western blot analysis with indicated antibodies. One representative blot of four independent culture batches is shown. (**B**) Total cellular RNAs were extracted and subjected to quantitative RT-PCR for the measurement of CD163 and arginase 1 mRNA expression. * *p* < 0.05 vs. untreated control and # *p* < 0.05 vs. LPS/IFN-γ control, *n* = 4.

The pro-inflammatory phenotype of microglia is closely linked with an increased expression of P2X purinoceptor 4 (P2X4R), P2X7R, P2Y12R, Inducible Nitric Oxide Synthase (iNOS), Cyclooxygenase 2 (COX-2), and CD68. Oppositely, the alternative or anti-inflammatory phenotype of microglia is associated with a high expression of CD163 and arginase 1 [16,17,22,31,32,33]. In neuron/glia cultures, LPS/IFN-γ elevated the protein levels of P2X4R, P2X7R, P2Y12R, iNOS, COX-2 (Figure 3A), and CD68 (Figure 2C) while had little effect on the mRNA levels of CD163 and arginase 1 (Figure 3B). The levels of reactive microglia-associated proteins were alleviated by β-funaltrexamine (Figure 2C and Figure 3A). However, β-funaltrexamine elevated mRNA expression in anti-inflammatory phenotype microglia-associated CD163 and arginase 1 (Figure 3B). These findings imply that microglia are targets to the actions of β-funaltrexamine and that the alleviation of pro-inflammatory and promotion of anti-inflammatory phenotypes of microglia may be attributed to the anti-inflammatory effects of β-funaltrexamine.

### 2.4. β-Funaltrexamine Alleviated Inflammatory Intracellular Signaling

**Figure 4:**
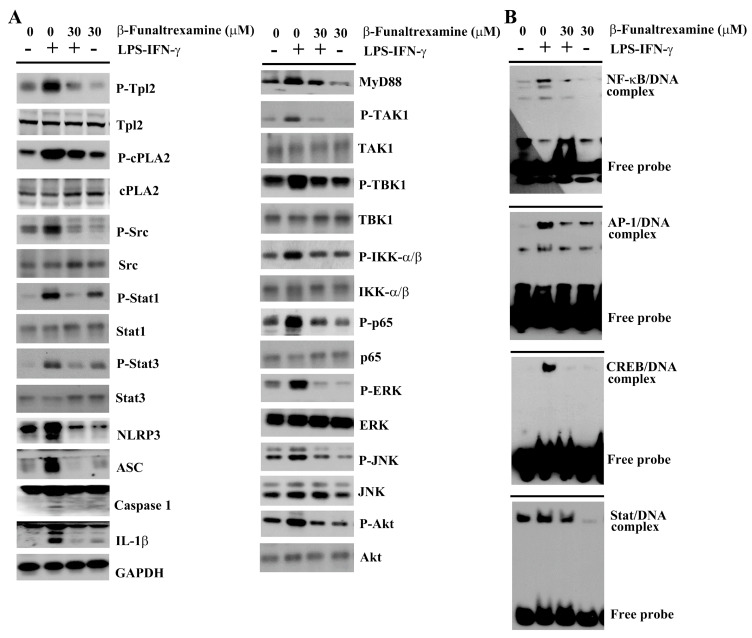
β-funaltrexamine alleviated intracellular signaling molecule activation. Neuron/glia cultures were pretreated with vehicle or β-funaltrexamine (30 μM) for 30 min before being incubated with LPS (100 ng/mL)/IFN-γ (10 U/mL) for an additional 4 h. (**A**) Total cellular proteins were extracted and subjected to Western blot analysis with indicated antibodies. (**B**) Nuclear proteins were extracted and subjected to Electrophoretic Mobility Shift Assay (EMSA) for the measurement of NF-κB, AP-1, cAMP-Response Element Binding Protein (CREB), and Stat DNA binding activities. One representative blot of four independent culture batches is shown.

**Figure 5:**
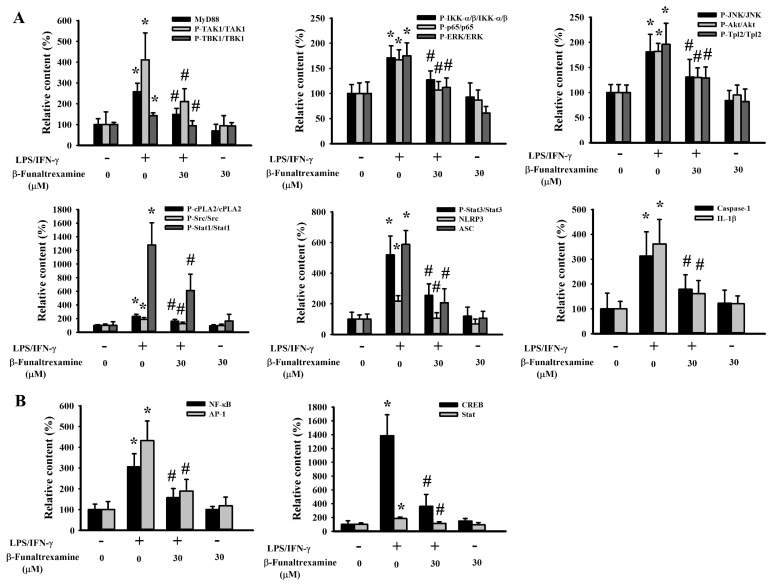
Quantitative data of β-funaltrexamine-alleviated intracellular signaling molecules: Neuron/glia cultures were pretreated with vehicle or β-funaltrexamine (30 μM) for 30 min before being incubated with LPS (100 ng/mL)/IFN-γ (10 U/mL) for an additional 4 h. (**A**) Total cellular proteins were extracted and subjected to Western blot analysis with indicated antibodies. (**B**) Nuclear proteins were extracted and subjected to EMSA for the measurement of NF-κB, AP-1, CREB, and Stat DNA binding activities. Quantitative data are shown. * *p* < 0.05 vs. untreated control and # *p* < 0.05 vs. LPS/IFN-γ control, *n* = 4.

The Toll-Like Receptor (TLR) axis commonly converges various endogenous and exogenous stimuli, including the LPS and IFN-γ, to intracellular signaling molecules and latent transcriptional factors critical to the acquisition of pro-inflammatory phenotypes of microglia and the transcriptional activation of pro-inflammatory cytokines [31,34]. Treatment of neuron/glia cultures with LPS/IFN-γ increased protein expression in MyD88, NLR Family Pyrin Domain Containing 3 (NLRP3), Apoptosis-Associated Speck-Like Protein Containing a CARD (ASC), protein phosphorylation in Transforming Growth Factor β-Activated Kinase-1 (TAK1), TANK-binding Kinase 1 (TBK1), IκB Kinase α/β (IKK-α/β), p65, Extracellular Signal-regulated Kinase (ERK), c-Jun N-terminal Kinase (JNK), Akt, Tumor Progression Locus 2 (Tpl2), cytosolic Phospholipase A2 (cPLA2), Src, Signal Transducers and Activators of Transcription 1 (Stat1), and Stat3 (Figure 4A and Figure 5A) as well as DNA binding activity in NF-κB, AP-1, cAMP-Response Element Binding Protein (CREB), and Stat (Figure 4B and Figure 5B). Besides, a cleavage of caspase-1 and IL-1β protein was found (Figure 4A and Figure 5A). In the presence of β-funaltrexamine, there was a decline in protein expression, protein phosphorylation, protein cleavage, and DNA binding activity (Figure 4 and Figure 5). These findings imply that the anti-inflammatory and neuroprotective effects of β-funaltrexamine may be attributed to its inhibition on the axis of TLRs, NF-κB, AP-1, CREB, and Stat signaling.

### 2.5. β-Funaltrexamine Alleviated Cerebral I/R Injury

**Figure 6:**
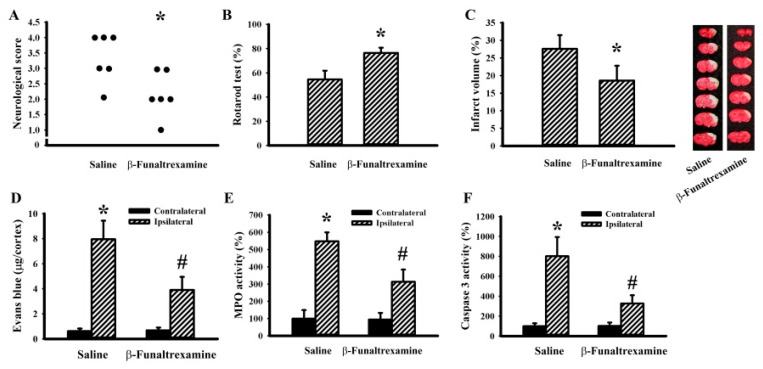
β-funaltrexamine protected against cerebral I/R injury. Rats receiving normal saline vehicle or β-funaltrexamine (82.5 nmol/30 μL) intracerebroventricular infusion were subjected to both Common Carotid Arteries (CCAs) and the right middle cerebral artery (MCA) occlusion for 90 min followed by 24-h reperfusion. (**A**) Neurological deficits were evaluated by neurological score. (**B**) The motor performance was assessed by a Rotarod test. (**C**) Representative photographs show the histological examination of a brain infarction by Triphenyltetrazolium Chloride (TTC) staining. The average percentage of infarction volume in the ipsilateral hemisphere is depicted. (**D**) The contents of Evans blue in contralateral and ipsilateral cortical tissues were measured by an Evans blue extravasation assay. Proteins were extracted from the contralateral and ipsilateral cortical tissues and subjected to an enzymatic assay of Myeloperoxidase (MPO) activity (**E**) and caspase 3 activity (**F**). * *p* < 0.05 vs. saline or the contralateral tissues of the vehicle groups and # *p* < 0.05 vs. the ipsilateral tissues of the vehicle groups, *n* = 6.

**Figure 7:**
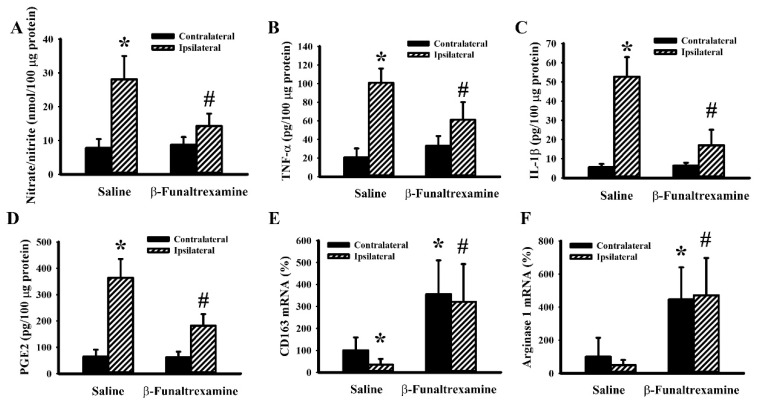
β-funaltrexamine alleviated cerebral I/R-induced neurotoxic cytokine production. Rats receiving normal saline vehicle or β-funaltrexamine (82.5 nmol/30 μL) intracerebroventricular infusion were subjected to both CCAs and the right MCA occlusion for 90 min followed by 24-h reperfusion. Total homogenates (ipsilateral and contralateral cortical tissues) were subjected to Griess reagent or ELISA for the measurement of NO (**A**), TNF-α (**B**), IL-1β (**C**), and PGE2 (**D**) content. Total cellular RNAs were isolated from the ipsilateral and contralateral cortical tissues and subjected to quantitative RT-PCR for the measurement of CD163 (**E**) and arginase 1 (**F**) mRNA content. * *p* < 0.05 vs. the contralateral tissues of the vehicle groups and # *p* < 0.05 vs. the ipsilateral tissues of the vehicle groups, *n* = 6.

To further explore the in vivo effects of β-funaltrexamine on neuroinflammation and neurodegeneration, a rat model showing cerebral I/R brain injury was established. Transient focal cerebral I/R caused neurological deficits (Figure 6A), impaired sensorimotor performance (Figure 6B), brain infarction (Figure 6C), Blood–brain Barrier (BBB) disruption (Figure 6D), neutrophil infiltration (Figure 6E), and cell apoptosis (Figure 6F). Intracerebroventricular β-funaltrexamine infusion improved postischemic changes (Figure 6). It was noted that a parallel reduction in reactive microglia released NO (Figure 7A), TNF-α (Figure 7B), IL-1β (Figure 7C), and PGE2 (Figure 7D) as well. The mRNA levels of anti-inflammatory microglia-associated CD163 (Figure 7E) and arginase 1 (Figure 7F) were elevated by β-funaltrexamine. Therefore, β-funaltrexamine displays protective potential against ischemic brain injury concurrently with the amelioration of microglia activation and neuroinflammation.

## 3. Discussion

Our groups have demonstrated that opioids modulate postischemic progression in a rat model experiencing stroke [11,24]. The studies presented here further extend earlier findings that the μ opioid receptor antagonist β-funaltrexamine possesses both anti-inflammatory and neuroprotective effects in vitro and in vivo. Using rat neuron/glia cultures, β-funaltrexamine protected against LPS/IFN-γ-induced microglia activation, neurotoxic cytokine expression, and neuronal cell death. β-funaltrexamine-mediated the downregulation of pro-inflammatory phenotype of microglia, the upregulation of anti-inflammatory phenotype of microglia, the reduction of neurotoxic cytokine expression, and the inhibition of neuronal cell death, which could be attributed to its interference in the TLR axis and NF-κB, AP-1, CREB, and Stat controlled transcriptional programs. Parallel anti-inflammatory and neuroprotective effects were demonstrated in a rat model experiencing cerebral I/R injury through an intracerebroventricular injection of β-funaltrexamine. These findings provide new insight into the anti-inflammatory and neuroprotective mechanisms of β-funaltrexamine in combating neurodegenerative diseases, such as stroke.

The interaction between the opioidergic and immune systems has been a long-term concern for healthcare professionals. Morphine induces potent analgesia and is commonly prescribed to patients with moderate-to-severe pain. However, the chronic use of morphine leads to tolerance and hyperalgesia due to its pro-inflammatory potential. There have been many studies performed over the years showing the pro-inflammatory mechanisms of morphine and the μ opioid receptor agonists in humans, rodents, and cells, including the microglia [13,14,15,16,17,18]. Contrary to the actions of the μ opioid receptor agonists, the agonists of δ and κ opioid receptors suppress inflammatory responses [20,21,22,23]. The opioid-immune crosstalk is further complicated by the findings that the nonselective opioid receptor antagonist (−)-naloxone and its inactive stereoisomer (+)-naloxone share both anti-inflammatory and neuroprotective effects under certain situations [3,11,13,18,24,25,26]. Currently, the opioid receptor-dependent and -independent anti-inflammatory mechanisms in the relevant studies have been described as being associated with the involvement of TLRs, P2XRs, Histone Deacetylase 6 (HDAC6), Protein Kinase C, Mitogen-Activated Protein Kinase (MAPKs), NF-κB, LPS binding, superoxide anion, and macrophages/microglia polarization.

Clinically, cancer patients undergoing intensive morphine treatment have an increased risk of stroke incidence [34]. Accordingly, a growing body of evidence has revealed the neuroprotective effects of the nonselective opioid receptor antagonist naloxone, δ opioid receptor agonists, and κ opioid receptor agonists in rodent models experiencing neurodegeneration [3,7,9,10,11,24,25,26,35,36,37]. Being a CNS-penetrating compound, the selective μ opioid receptor antagonist β-funaltrexamine is capable of inhibiting LPS-induced neuroinflammation in mice after receiving an intraperitoneal injection [29,30]. Its anti-inflammatory actions are further demonstrated in human astroglial cells under distinct stimuli, including LPS, HIV-1 Tat, and IL-1β. The inhibition of iNOS and chemokine expression is mediated through μ opioid receptor independent mechanisms and involves p38 and NF-κB pro-inflammatory cascade [19,27,28,29,30]. Through intracerebroventricular infusion, we had described the neuroprotective potential of β-funaltrexamine against cerebral I/R injury in rats [24]. Herein, extended studies surrounding anti-neuroinflammation and neuroprotection were demonstrated in rat neuron/glia cultures and a rat model with cerebral I/R injury, while highlighting microglia phenotypes as being targets of β-funaltrexamine’s actions. Despite the distinct insults in vitro or in vivo, we found that the presence of β-funaltrexamine downregulated the pro-inflammatory phenotype of microglia, reduced neurotoxic cytokine expression, and upregulated the anti-inflammatory phenotype of microglia. Our findings imply that β-funaltrexamine may act as an anti-inflammatory agent against various inflammatory insults.

As with macrophages, microglia adopt a series of molecular and biochemical events to switch genetic programs, morphological cytoskeletons, cell membrane surface presentation, and functional outcomes. The rising of pro-inflammatory and the subsiding of anti-inflammatory phenotypes of microglia have been implicated in various neurodegenerative diseases as well as a reversal in the balance of ameliorate disease progression [38,39,40,41]. Typically, neurotoxic cytokines such as TNF-α, IL-1β, IL-18, IL-6, IFN-γ, NO, PGE2, and free radicals come from classical activation polarized microglia. Microglia, in the alternative activation polarization, release Transforming Growth Factor (TGF-β), IL-4, and IL-10, with the consequences of suppressing inflammation and restoring homeostasis. The presence of P2X4R, P2X7R, and P2Y12R promote microglia polarization towards the classical activation state, while CD163 and arginase 1 promote polarizing microglia towards the alternative activation state [32,38,39,40,41,42]. Alterations in microglia polarization favoring the classical activation state occurred in LPS/IFN-γ-stimulated neuron/glia cultures and cerebral I/R-injured cortical brains. β-funaltrexamine shifted the polarization of microglia towards the anti-inflammatory phenotype from the pro-inflammatory, as evidenced by a decrease in NO, TNF-α, IL-1β, and PGE2 as well as increased CD163 and arginase 1. In a cell model of neuron/glia cultures, the effects of β-funaltrexamine were further supported by decreasing the expression of polarization transcription factors and regulators, including P2X4R, P2X7R, and P2Y12R. Although the microglia polarization-associated regulators and the detailed microglia phenotypes were not completely investigated, current findings highlight that the reversal of microglia pro-inflammatory polarization and a switch towards the alternative activation state may be an action mechanism of β-funaltrexamine done to ameliorate neuroinflammation.

Upon cerebral I/R injury or LPS/IFN-γ stimulation, the transmembrane TLR4 represents an active player to engage with various exogenous and endogenous immunocompetent ligands. It can then turn on and spread signaling networks and eventually impact on the nuclear influx activity, DNA binding activity, and transcriptional activity of transcription factors, including NF-κB, AP-1, CREB, and Stat [34,42]. Likewise, the engagement of IFN-γ with receptors activates Stat1 to drive Interferon Regulatory Factor 1 (IRF1) expression, by which transcription factors Stat1 and IRF1 work in concert to boost another wave of transcriptional program. Additionally, the activation of the Stat family latent transcriptional factors is promoted by upstream stimulatory tyrosine kinases such as the Janus Kinase (Jak) and Src family kinases [31,34]. A vast array of linking adaptors and signaling molecules, including MyD88, TAK1, TBK1, Tpl2, cPLA2, ERK, JNK, IKK-α/β, p65, Src, as well as Akt, will have their expression or phosphorylation elevated in LPS/IFN-γ-stimulated neuron/glia cultures. The activation of inflammasome (NLRP3, ASC, and caspase-1) and IL-1β production can boost another wave of pro-inflammatory signaling. Our previous studies indicate that pharmacological inhibitors corresponding to several steps of LPS/IFN-γ intracellular actions caused a reduction in both the production of neurotoxic mediators and neuronal cell death in cultured neuron/glia cultures [31]. β-funaltrexamine alleviated LPS/IFN-γ-activated downstream adaptors, signaling molecules, and transcription factors. Other than microglia, β-funaltrexamine downregulated HIV-1 Tat-, LPS-, and IL-1β-induced NF-κB activation and chemokine expression in human astroglial cells [19,27,28,29,30]. These phenomena highlight the role of β-funaltrexamine in suppressing intracellular pro-inflammatory signaling.

The endogenous opioidergic system is widely distributed throughout the CNS and periphery, while also linking with many physiological activities. The shared and distinct post-receptor consequences from the μ, δ, and κ receptors not only highlight but also complicate their roles in disease initiation and progression. Currently, their crosstalk with the immune system is of clinical attraction. The development of κ opioid receptor mimetic and μ opioid receptor antagonists based on β-funaltrexamine and the μ opioid receptor complex are continuing to find neuroactive candidates [43,44]. Through this study, we have provided experimental evidence showing the anti-inflammatory and neuroprotective effects of β-funaltrexamine for neurodegenerative diseases in rat neuron/glia cultures as well as a rat model with cerebral I/R injury. The effects of β-funaltrexamine are closely linked with its actions on microglia by switching microglia polarization from pro-inflammatory towards anti-inflammatory phenotypes. However, its effects on CNS cell types other than microglia have yet to be explored. Chronic treatment with morphine increases the risk of stroke incidence, and rodent studies have indicated its effects on the blood–brain barrier breakdown involving the μ oipoid receptor [25,45]. Although there still remain limitations to our experiments, the CNS permeable β-funaltrexamine is a proposed anti-inflammatory and neuroprotective candidate for the treatment of neuroinflammation-accompanied neurodegenerative diseases such as stroke. Before it can be translated into clinical practice, however, a deeper investigative insight into its peripheral administration and CNS actions is still required.

## 4. Materials and Methods

### 4.1. Cell Cultures

The Animal Experimental Committee of Taichung Veterans General Hospital reviewed and approved the protocols for preparation of primary neuron/glia and microglia cultures from the cerebral cortices of postnatal day 1 Sprague–Dawley rats (La-101986, 29 October 2012). Briefly, the dissected cortices were dissociated by physical trituration and enzymatic digestion, with the dissociated cells then plated onto poly-d-lysine-coated dishes in accordance with previously reported methods [31]. Neuron/glia was prepared by maintaining the cells in a minimum essential medium, supplemented with 10% Fetal Bovine Serum (FBS) and 10% horse serum for 10–12 days. The specific cell types were identified by immunofluorescence staining with antibodies recognizing neurons (MAP-2, Transduction Laboratories, Franklin Lakes, NJ, USA), astrocytes (GFAP, Santa Cruz Biotechnology, Dallas, TX, USA), and microglia (CD68, Santa Cruz Biotechnology). Neuron/glia consisted of 30–40% neurons, 40–50% astrocytes, and 10–15% microglia. The murine microglia BV2 cell line and macrophage RAW264.7 cell line were maintained in Dulbecco’s Modified Eagle Medium (DMEM) containing 10% FBS [33].

### 4.2. Cytotoxicity Assessment

The cytotoxicity was assessed based upon the efflux of LDH to the culture media using the Pierce^TM^ LDH Cytotoxicity Assay Kit (Thermo Fisher Scientific, Waltham, MA, USA). The levels of cytotoxicity were expressed as the ratio of released LDH and total LDH.

### 4.3. Immunofluorescence Staining

Prior to antibody reaction, neuron/glia (24-well plates) were fixed with 4% paraformaldehyde, permeabilized with 0.1% Triton X-100, and blocked with 5% nonfat milk. Then, the cells were incubated with antibodies against MAP-2, GFAP, or CD68 and followed by Fluorescein Isothiocyanate (FITC)-conjugated secondary antibody. The cell nuclei were counterstained with Hoechst 33342. The immunofluorescence signals were visualized using a fluorescence microscope. For the measurement of MAP-2-, CD68-, and GFAP-positive cell numbers, the total numbers of immunopositive cells were counted from randomly selected four fields in a well of 24-well plates. Four replicates were done for each experiment.

### 4.4. Enzyme-Linked Immunosorbent Assay (ELISA)

The cell cultured supernatants (100 μL) and brain cortical protein extracts (100 μg) were subjected to the measurement of TNF-α, IL-1β, and PGE2 using commercial ELISA kits (R&D Systems, Minneapolis, MN, USA). For HNO_2_^−^/HNO_3_^−^ (nitrite/nitrate) determination, the samples were subjected to the measurement using a Griess Reagent Kit (Thermo Fisher Scientific).

### 4.5. Western Blot Analysis

Proteins were extracted from cells and the dissected cortical tissues using the commercial Tissue Protein Extraction Reagents (T-PER, Pierce Biotechnology, Rockford, IL, USA). Proteins went through SDS-PAGE separation and electrical transfer, with the membranes sequentially incubated with primary antibodies, horseradish peroxidase-labeled IgG, and enhanced chemiluminescence Western blotting reagents. The visualized signals were quantitated using a computer image analysis system (IS1000; Alpha Innotech Corporation, San Leandro, CA, USA). Proteins recognized by the primary antibodies were MyD88 (sc-136970), TAK1 (sc-7967), phospho-TAK1(sc-130219), TBK1 (sc-52957), phospho-TBK1 (sc-130219), IKK-α/β (sc-7607), phospho-IKK-α/β (sc-293135), p65 (sc-8008), phospho-p65 (sc-166748), ERK (sc-514302), phospho-ERK (sc-7383), JNK (sc-7345), phospho-JNK (sc-6254), Akt (sc-8312), phospho-Akt (sc-7985-R), Src (sc-5266), phospho-Src (sc-166860), Stat1 (sc-8394), phospho-Stat1 (sc-136229), Stat3 (sc-8019), phospho-Stat3 (sc-8059), P2X4R (sc-15190), P2X7R (sc-514962), P2Y12R (sc-27152), CD68 (sc-20060), iNOS (sc-651), COX-2 (sc-19999), GFAP (sc-33673), cPLA2 (sc-454), Glyceraldehyde 3-Phosphate Dehydrogenase (GAPDH) (sc-32233), ASC (sc-271054), caspase-1 (sc-392736) (Santa Cruz Biotechnology), phospho-cPLA2 (#2831, Cell Signaling, Danvers, MA, USA), MAP-2 (sc-74421) (Transduction Laboratories), Tpl2 (sc-720), phospho-Tpl2 (sc-1717) (R&D Systems), NLRP3 (sc-134306), and IL-1β (sc-52012)(Thermo Fisher Scientific).

### 4.6. RNA Isolation and Quantitative Real-Time Reverse Transcriptase Polymerase Chain Reaction (RT-PCR)

The total RNAs were extracted from the cells and cortical tissues and subjected to the cDNA synthesis and real-time PCR (ABI StepOne^TM^, Applied Biosystems, Foster City, CA, USA) according to our previously reported methods [42]. The levels of mRNA expression were calculated using the ΔΔC_t_ method and normalized with β-actin. Oligonucleotide sequences used in PCR were 5’-CCAGTCCCAAACACTGTCCT and 5’-ATGCCAGTGAGCTTCCCGTTCAGC for CD163; 5’-GGAATCTGCATGGGCAACCTGTGT and 5’AGGGTCTACGTCTCGCAAGCCA for arginase 1; and 5′-AAGTCCCTCACCCTCCCAAAAG and 5′-AAGCAATGCTGTCACCTTCCC for β-actin.

### 4.7. Preparation of Nuclear Extracts and Electrophoretic Mobility Shift Assay (EMSA)

Nuclear proteins were extracted from the cells and dissected cortical tissues using a commercially available nuclear extraction kit and an EMSA kit (Panomics, Fremont, CA, USA). As with our previous studies [31], nuclear extracts (5 μg) were reacted with AP-1 oligonucleotide (5′-CGCTTGATGAGTCAGCCGGAA), NF-κB oligonucleotide (5′-AGTTGAGGGGACTTTCCCAGGC), Stat oligonucleotide (5′-ATCGTTCATTTCCCGTAAATCCCTA), or CREB oligonucleotide (5′-AGAGATTGCCTGACGTCAGAGAGCTAG). The DNA/protein complexes in the membranes were visualized by enhanced chemiluminescence Western blotting reagents and quantitated using a computer image analysis system (IS1000; Alpha Innotech Corporation).

### 4.8. Cerebral I/R

The Animal Experimental Committee of Taichung Veterans General Hospital reviewed and approved the protocols involving animal studies, while strictly adhering to the institute’s guidelines (La-101986, 29 October 2012). Transient focal cerebral I/R was produced on adult male Sprague–Dawley rats (300 ± 20 g, *n* = 36 in total) by occluding the two Common Carotid Arteries (CCA) and the right Middle Cerebral Artery (MCA) for 90 min, followed by reperfusion for 24 h [11]. Under anesthesia with isoflurane (2–4%), the rat’s head was mounted in a stereotaxic apparatus and the skull was then partially removed to show the MCA. Pharmacological agents were infused through a cannula inserted perpendicularly into the right lateral ventricle anteroposteriorly at −0.9 mm, mediolaterally at 1.5 mm from the Bregma, and dorsoventrally at −3.5 mm from the dural surface. An intracerebroventricular infusion was initiated 1 h prior to the occlusions and continued for a total of 4 h. The animals were sacrificed 24 h after behavioral evaluation.

### 4.9. Quantification of Ischemic Infarction

Under anesthesia with isoflurane (2–4%), the rats (*n* = 6 per group) were decapitated. The brains were cut into a serial coronal section at a 2-mm interval from the frontal pole. The slices were then immersed in 2% Triphenyltetrazolium Chloride (TTC) solution at 37 °C for 30 min, followed by fixation in a 10% phosphate-buffered formalin for 45 min [11]. Brain infarction was highlighted by a white color, and the volume was measured with a computer image analysis system (IS1000; Alpha Innotech Corporation).

### 4.10. Evans Blue Extravasation Assay

Under anesthesia with isoflurane (2–4%), rats (*n* = 6 per group) were injected with Evans blue (4%, 1 mL/kg) via the tail vein 3 h prior to being sacrificed. Following perfusing with heparinized saline solution, the rats were decapitated and the ipsilateral and contralateral cortices were isolated. The tissues were homogenized with Phosphate-Buffered Saline (PBS) and precipitated with Trichloroacetic Acid (TCA) (100%). The contents of Evans blue in the supernatants were measured in a photometer (absorbance at 620 nm) and calculated according to a standard curve.

### 4.11. Neurological Evaluation

Technicians who had been blind to the treatment evaluated the sensorimotor performances using a modified six-point neurological deficit severity scale (*n* = 6 per group) [42]. Neurological evaluation was performed prior to animal sacrifice, as follows: 0: no neurological deficit; 1: difficulty in fully extending the left forepaw; 2: unable to extend the left forepaw; 3: mild circling to the left; 4: severe circling to the left; and 5: falling to the left.

### 4.12. Rotarod Test

Motor coordination was evaluated through use of a Rotarod test [46]. Rats (*n* = 6 per group) were placed on the Rotarod cylinder with speed increasing from 4 rpm to 40 rpm within 5 min time, with the time that the animals remained on the Rotarod cylinder being measured. The mean duration time on the device was recorded with three Rotarod measurements. A percentage of mean duration on the Rotarod cylinder before sacrifice as well as each basal control (before occlusion) were calculated.

### 4.13. Myeloperoxidase (MPO) Activity Assay

Under anesthesia with isoflurane (2–4%), rats (*n* = 6 per group) were decapitated and the ipsilateral and contralateral cortices were isolated. Proteins were extracted and subjected to MPO activity measurement using a Myeloperoxidase (MPO) Activity Assay Kit (Abcam, ab105136), according to the manufacturer’s instructions.

### 4.14. Caspase 3 Activity Assay

Under anesthesia with isoflurane (2–4%), rats (*n* = 6 per group) were decapitated and the ipsilateral and contralateral cortices were isolated. Proteins were extracted and subjected to enzymatic measurement of caspase 3 activity using a commercial fluorometric protease assay kit (BioVision, Mountain View, CA, USA).

### 4.15. Statistical Analysis

All statistical results are presented as mean ± standard deviation. A one-way analysis of variance (ANOVA) was performed to evaluate experimental values between groups, with a consequent Dunnett’s test or Tukey post–hoc test performed for the purpose of comparison. It was considered statistically significant when the *p* value was less than 0.05.

## Abbreviations

ASC	Apoptosis-Associated Speck-Like Protein Containing a CARD
BBB	Blood–Brain Barrier
CCA	Common Carotid Arteries
CNS	Central Nervous System
COX-2	Cyclooxygenase 2
cPLA2	cytosolic Phospholipase A2
DMEM	Dulbecco’s Modified Eagle Medium
ELISA	Enzyme-Linked Immunosorbent Assay
EMSA	Electrophoretic Mobility Shift Assay
ERK	Extracellular Signal-regulated Kinase
FBS	Fetal Bovine Serum
FITC	Fluorescein Isothiocyanate
GADPH	Glyceraldehyde 3-Phosphate Dehydrogenase
GFAP	Glial Fibrillary Acidic Protein
IFN-γ	Interferon-γ
IL-1β	Interleukin-1β
IKK-α/β	IκB Kinase α/β
iNOS	Inducible Nitric Oxide Synthase
I/R	Ischemia/Reperfusion
IRF1	Interferon Regulatory Factor 1
JNK	c-Jun N-terminal Kinase
LDH	Lactate Dehydrogenase
LPS	Lipopolysaccharide
MAP-2	Microtubule-associated Protein 2
MCA	Middle Cerebral Artery
MPO	Myeloperoxidase
NLRP3	NLR Family Pyrin Domain Containing 3
NO	Nitric Oxide
P2X4R	P2X purinoceptor 4
PGE2	Prostaglandin E2
RT-PCR	Reverse Transcriptase Polymerase Chain Reaction
Stat1	Signal Transducers and Activators of Transcription 1
TAK1	Transforming Growth Factor β-Activated Kinase-1
TBK1	TANK-binding Kinase 1
TCA	Trichloroacetic Acid
TLR	Toll-Like Receptor
TNF-α	Tumor Necrosis Factor-α
Tpl2	Tumor Progression Locus 2
